# A versatile bulk electrotransfection protocol for murine embryonic fibroblasts and iPS cells

**DOI:** 10.1038/s41598-020-70258-w

**Published:** 2020-08-07

**Authors:** Shahin Eghbalsaied, Iqbal Hyder, Wilfried A. Kues

**Affiliations:** 1 Department of Biotechnology, Friedrich-Loeffler-Institut (FLI), Höltystr. 10, 31535 Neustadt, Germany; 2 Department of Animal Science, Isfahan (Khorasgan) Branch, Islamic Azad University, Isfahan, Iran

**Keywords:** Biological techniques, Biotechnology, Cell biology

## Abstract

Although electroporation has been widely accepted as the main gene transfer tool, there is still considerable scope to improve the electroporation efficiency of exogenous DNAs into primary cells. Here, we developed a square-wave pulsing protocol using OptiMEM-GlutaMAX for highly efficient transfection of murine embryonic fibroblasts (MEF) and induced pluripotency stem (iPS) cells using reporter genes as well as gRNA/Cas9-encoding plasmids. An electrotransfection efficiency of > 95% was achieved for both MEF and iPS cells using reporter-encoding plasmids. The protocol was efficient for plasmid sizes ranging from 6.2 to 13.5 kb. Inducing the error prone non-homologous end joining repair by gRNA/Cas9 plasmid transfection, a high rate of targeted gene knockouts of up to 98% was produced in transgenic cells carrying a single-copy of Venus reporter. Targeted deletions in the Venus transgene were efficiently (up to 67% deletion rate) performed by co-electroporation of two gRNA-encoding plasmids. We introduced a plasmid electrotransfection protocol which is straight-forward, cost-effective, and efficient for CRISPRing murine primary cells. This protocol is promising to make targeted genetic engineering using the CRISPR/Cas9 plasmid system.

## Introduction

The CRISPR (clustered regularly interspaced short palindromic repeats)-Cas9 (CRISPR-associated protein 9) nuclease system is a straight-forward, versatile, and highly efficient tool for genome editing of various organisms. Using ‘all-in-one’ expression vectors containing expression cassettes for guide RNAs and Cas9 nuclease/nickase it is possible to conduct CRISPR/Cas9 studies through a low-cost and straight-forward approach^1^. However, the efficiency of gene editing using plasmid-based delivery methods remains relatively low, which subsequently increased attention toward using alternate methods employing the Cas9 protein and/or guideRNA (gRNA), or using ribonucleoproteins (RNPs)^2,3^.

Basically, gene transfer into cells can be achieved via electroporation, lipofection, or viral transduction. Although viral gene transfer is very efficient, it requires time, skilled staff, and high levels of safety issues, whereas it has a limitation in the cargo size^4^. On the other side, lipofection suffers from a low efficiency in primary cells. Electroporation is an approach to instantly create pores in the cell membrane using a burst electric pulse and to mediate the transfer of micro- and macro-molecules into cells, embryos, tissues, and organs^5^. Since the early papers on electroporation^6^, it has been evident that gene transfer via electroporation is simple, easily applicable, and also efficient compared to lipofection^6^. Although electroporation has been widely accepted as the main gene transfer tool, the underlying mechanism has not been completely understood^7^. Therefore, optimization of various factors, such as the electroporation medium, cuvette type (path width, and length), as well as the pulsing method which includes the amount, number, duration, and interval of pulses is needed to have a high electrotransfection efficiency particularly in primary cells. Based on the above-mentioned electroporation parameters various types of electroporation-based devices, such as nucleofection^8^, nano-electroporation^9^, mechanical–electrical approach^7^, and microfluidic membrane deformation^2^ as well as new generation of electroporator devices^10^, have been invented. However, still there is a huge room to improve the electroporation efficiency to transfer exogenous DNAs into primary cells, specifically for large plasmids^7,11^.

Here, we introduced a highly efficient electrotransfection method for both murine embryonic fibroblast (MEF) and induced pluripotent stem (iPS) cells based on the square-wave pulsing method using a Bio-Rad electroporation device. We developed the technology using different types of reporter plasmids and confirmed its high efficiency for making gene knockouts (KO), which were induced by both insertion-deletions (indels) and targeted deletions using a combination of two gRNAs through CRISPR/Cas9 plasmids, respectively.

## Results

### Optimization of electrotransfection protocol for iPS and MEF cells

**Figure 1:**
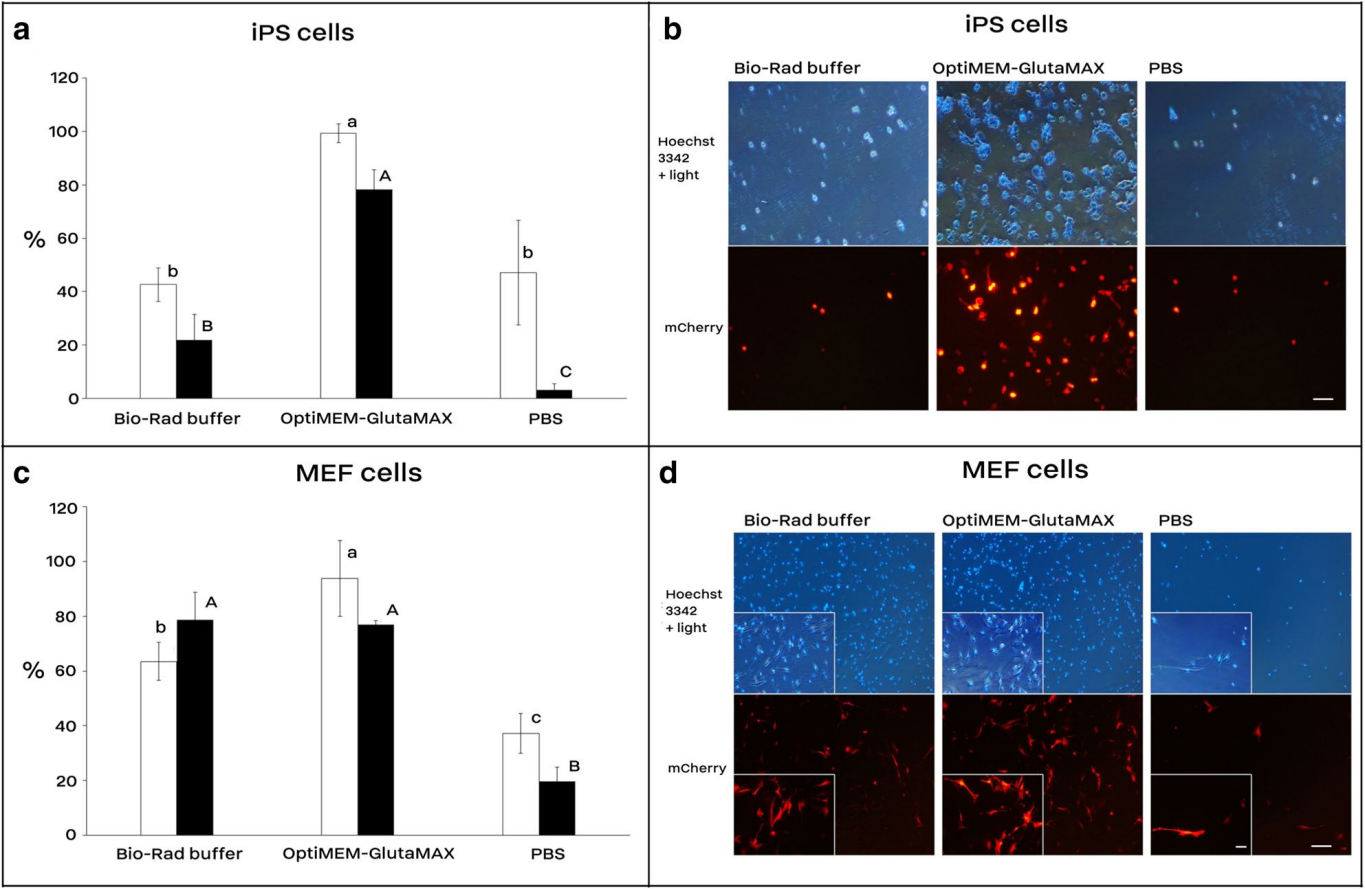
Cell electrotransfection and viability rates using Bio-Rad buffer, OptiMEM-GlutaMAX, and PBS. Electrotransfection efficiency of mouse iPS (**a** and **b**) and MEF cells (**c** and **d**). In each electroporation reaction, 20 µg (1.5–2.5 µg/µl) of the reporter plasmid (encoding mCherry) was pre-mixed with cells and underwent electroporation using the square-wave protocol consisted of 250 V for iPS and 300 V for MEF cells, 2 pulses, each 10 ms length, 10 s interval, and 4 mm cuvette. The reporter expression was assessed 36 h after electroporation under a fluorescence microscope. White and black bars are electrotransfection efficiency and cell viability, respectively. Bars with different A, B, C or a, b, c letters are significantly different (*p* value < 0.05). Scale bar is 100 µm; for MEF cells, a higher magnified photo is indented in the left part of each image with a scale bar of 5 µm. Results are means and standard deviation (n > 3).

**Figure 2:**
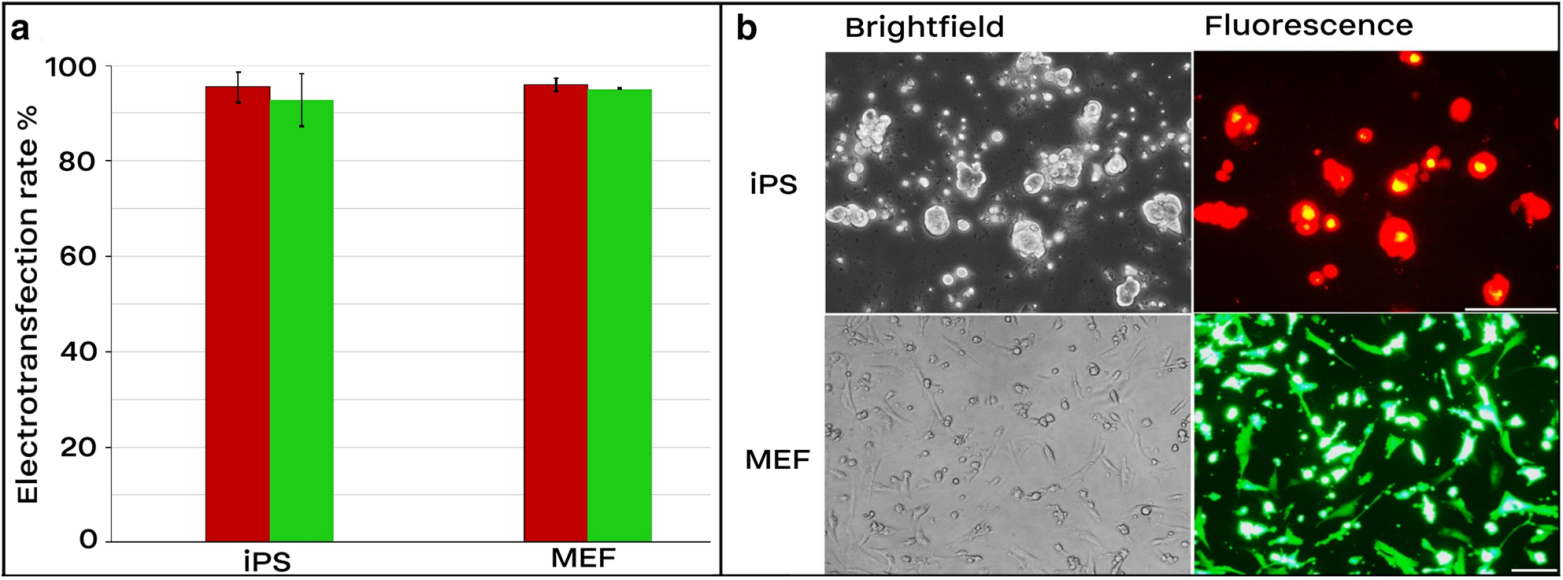
Electrotransfection efficiency of MEF and iPS cells. In each electroporation reaction, 20 µg (1.5–2.5 µg/µl) of either pT2-Venus and pT2-mCherry which encode Venus and mCherry proteins, respectively, were pre-mixed with cells and underwent electroporation. The following electroporation program was used: square-wave protocol with either 250 V for iPS or 300 V for MEF cells, each 10 ms pulse length, 2 pulses, 10 s pulse interval, and 4 mm cuvette. (**a**) Transfection efficiency. Transfected cells for mCherry and Venus are depicted by red and green bars, respectively. (**b**) Expression of Venus and mCherry 36 h after electroporation. Scale bar for iPS cells equals 100 µm. Scale Bar in fibroblasts is 10 µm. Results are means and standard deviation (n > 3).

We developed an electroporation protocol using the square-wave pulsing program of 250 V for iPS cells and 300 V for MEF cells, 2 pulses, each 10 ms length, and 10 s interval in 4 mm cuvettes (Fig. 1). Achieving this protocol, we optimized several parameters, from which electroporation media and pulse number and duration as well as the medium temperature are depicted in Supplementry Figures S1 and S2. Using the optimized electrotransfection protocol the transient transfection efficiency of murine iPS and MEF cells was assessed in 250 µl of either of Bio-Rad, OptiMEM-GlutaMAX, and PBS media (Fig. 1). Viability of iPS cells was significantly lower in Bio-Rad (22.0%) and PBS (3.2%) compared to the OptiMEM-GlutaMAX medium (78.2%). Also, the electrotransfection efficiency of iPS cells was more than two-fold higher in OptiMEM-GlutaMAX (99.3%) than that of Bio-Rad (42.7%) and PBS media (47.2%). The electrotransfection efficiency of MEF cells was 30% higher in OptiMEM-GlutaMAX compared to the Bio-Rad medium (*p* value < 0.05). In addition, the viability of both iPS and MEF cells were > 75% in the OptiMEM-GlutaMAX group. The same electrotransfection efficiency (92–96%) was achieved using mCherry- and Venus-encoding plasmids in both iPS and MEF cells (Fig. 2).

### Efficient knockout of Venus transgene using indels by Cas9/gRNA encoding plasmids

**Figure 3:**
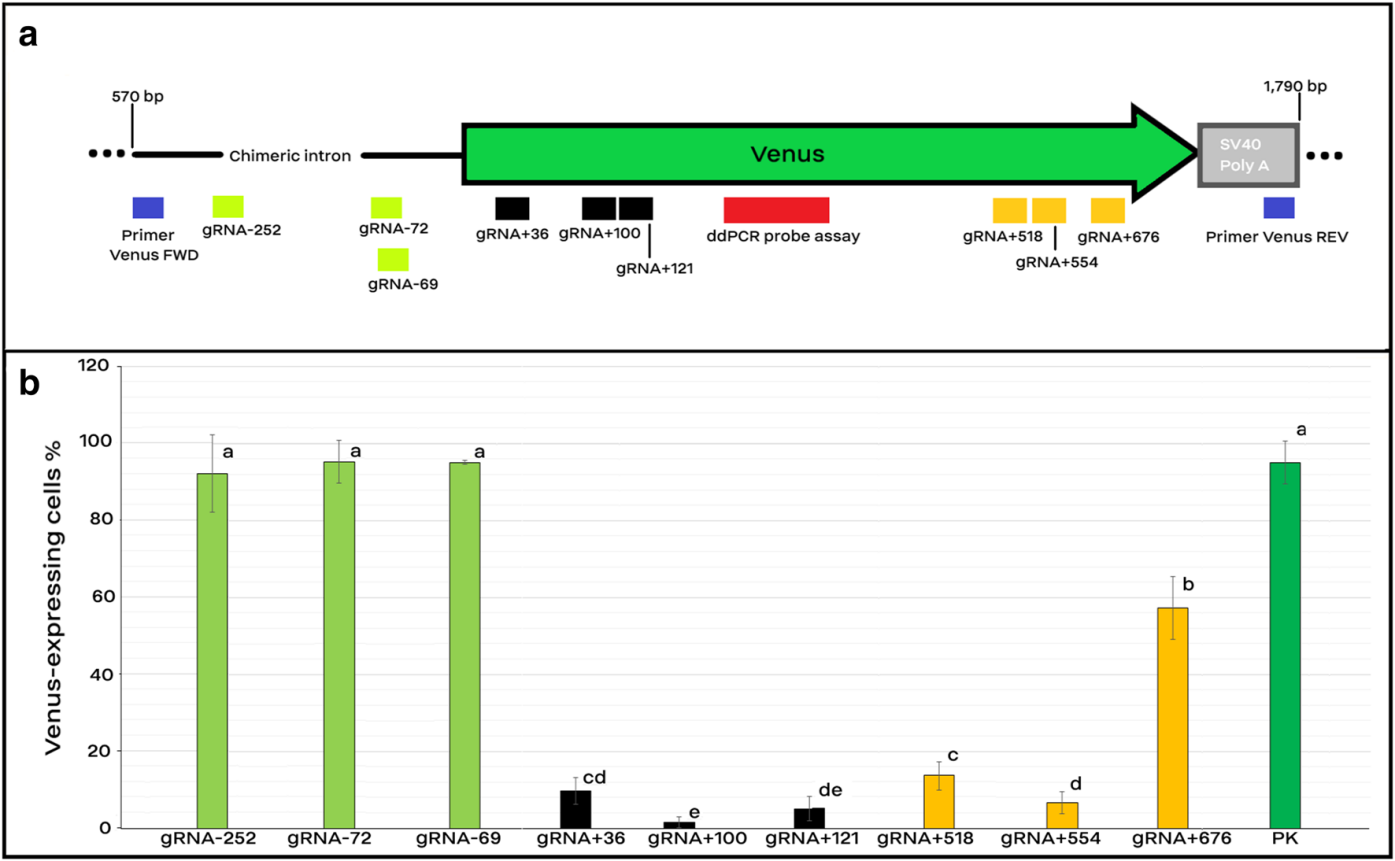
Target site and knockout efficiency of gRNAs. (**a**) Schematic presentation of gRNA target location on the Venus transgene. Nine gRNAs were designed to target the promoter region (− 252, − 72, and − 69), 5′ region (36, 100, and 121), and 3′ region (518, 554, and 676) of Venus transgene which are depicted with white-green, black, and yellow bars, respectively. The target site of the digital PCR assay is depicted in a red box. (**b**) Efficiency of different gRNAs for making Venus knockout. Cells were treated with puromycin and were screened for the Venus transgene under a fluorescence microscope 10 days after the electroporation (n > 3). Bars with common letters are not significantly different (p-value < 0.05). Cells with one copy of Venus, which did not undergo any electroporation treatment, were considered as the positive control for the Venus expression (PK).

**Figure 4:**
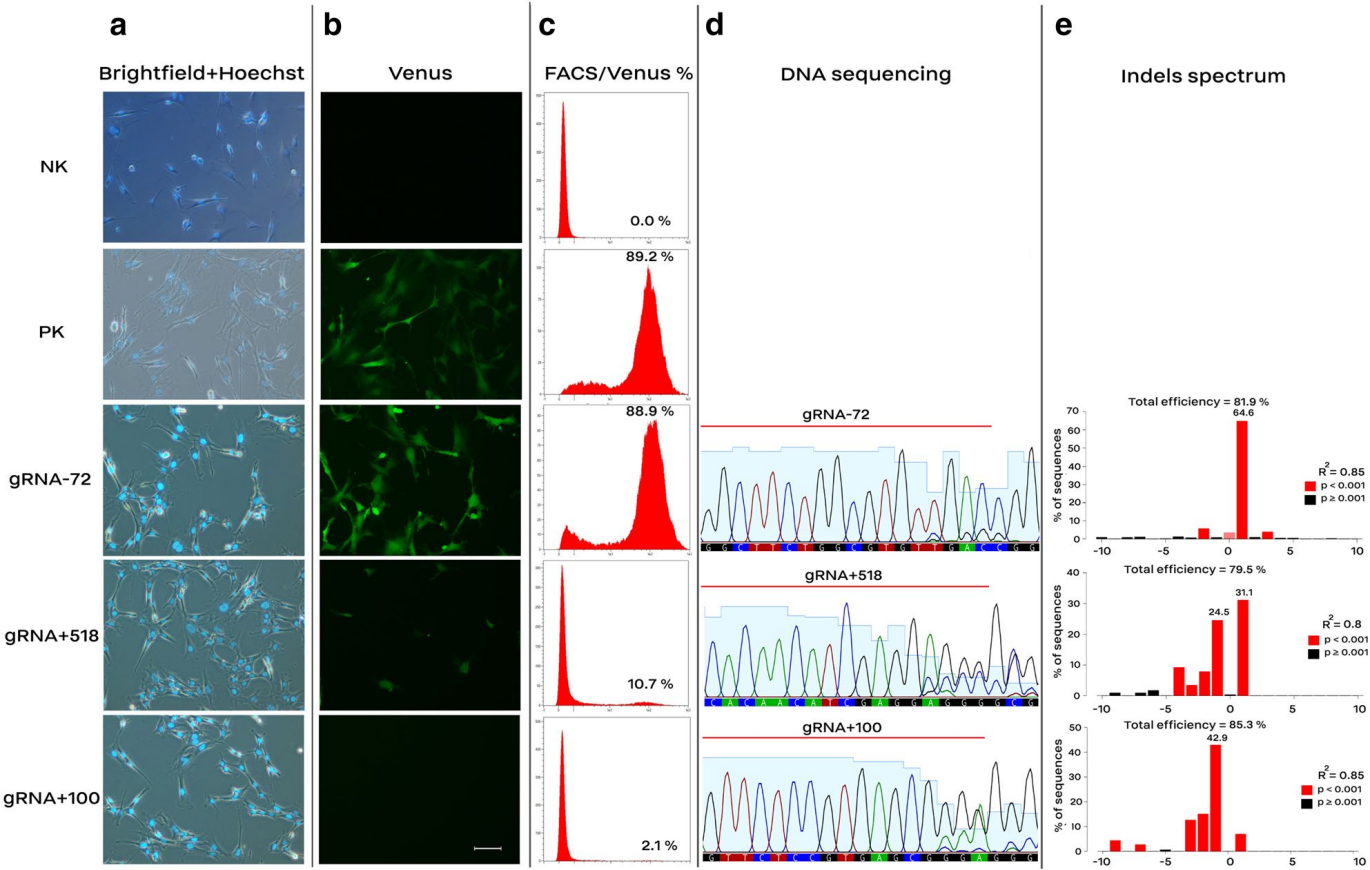
Knockout of Venus transgene by inducing indels using gRNA-encoding plasmids. MEF cells carrying a single-copy of the Venus transgene were electrotransfected with different gRNA-encoding plasmids (9.1 kb length). Induction of indels using gRNA-72 had no effect on the Venus expression, whereas gRNA + 100 resulted in loss of the Venus signal. Electroporated cells were selected against puromycin and screened 10 days after the electroporation. (**a** and **b**) Cells were stained with Hoechst 33342 and the efficiency of Venus knockout was assessed. Scale bar equals 10 µm. (**c**) Histoplots of the FACS results. (**d**) Partial sequencing results of the amplified Venus transgene. Target sites of the respective gRNAs are indicated by a red line, the first three nucleotides of the electropherogram are cropped to fit the image into the column. (**e**) Indels spectrum calculated by online tool for TIDE analysis. Cells with no copy of Venus were considered as the negative control (NK), and cells carrying a copy of Venus, without any electroporation treatment, were considered as the positive control for the Venus expression (PK). The following electroporation program was used: square-wave protocol: 20 µg plasmid (1.5–2.5 µg/µl), 300 V, 2 pulses, each 10 ms pulse length, 10 s pulse interval, and 4 mm cuvette (n > 3).

MEF cells carrying a single-copy of the Venus transgene were transfected by plasmids encoding a gRNA and Cas9 protein using the optimized electroporation method (Fig. 3). Venus expression was not reduced by three gRNAs which targeted the promoter region of the Venus transgene although they induced indels in the targeted sites (Figs. 3 and 4). However, targeting the 5′- and ending 3′-regions of the transgene, ranging from + 36 to + 554 bp of the cDNA, could knockout the transgene in > 90% of the electroporated cells (Fig. 3). The Venus KO efficiency was maximized using gRNA + 100 which was complementary to the 5′-region of the cDNA, with only 2% of cells maintained the functional Venus. The Venus knockout results were confirmed by fluorescence microscopy, as well as FACS analysis and DNA sequencing (Fig. 4C, D). The indels spectrum was obtained using the TIDE software (Fig. 4E). Since the pX459 plasmid encodes a puromycin resistant gene, we treated electrotransfected cells 24 h after the electroporation with the puromycin. The knockout efficiency of Venus transgene was not affected by the puromycin treatment; 99% vs. 93% for gRNA + 100 with and without puromycin selection, respectively (Supplementary Figure S3). Although electrotransfection results were promising using OptiMEM-GlutaMAX medium, substituting the medium with the standard OptiMEM supplemented with glutamine was inefficient for making Venus KO (Supplementary Figure S4). Moreover, electroporation of MEF cells with a large plasmid, pSGD-Lys-72 plasmid (13.5 kb), followed by a one-day selection against the puromycin antibiotic resulted in a similar cell viability with the pX459-gRNA-72 (9.2 kb) (Supplementary Figure S5). Both pSGD-Lys-72 and pX459-gRNA-72 encoded the Cas9 protein, gRNA-72, and a puromycin resistant gene.

### Highly efficient deletion of Venus fragments using dual gRNAs

**Figure 5:**
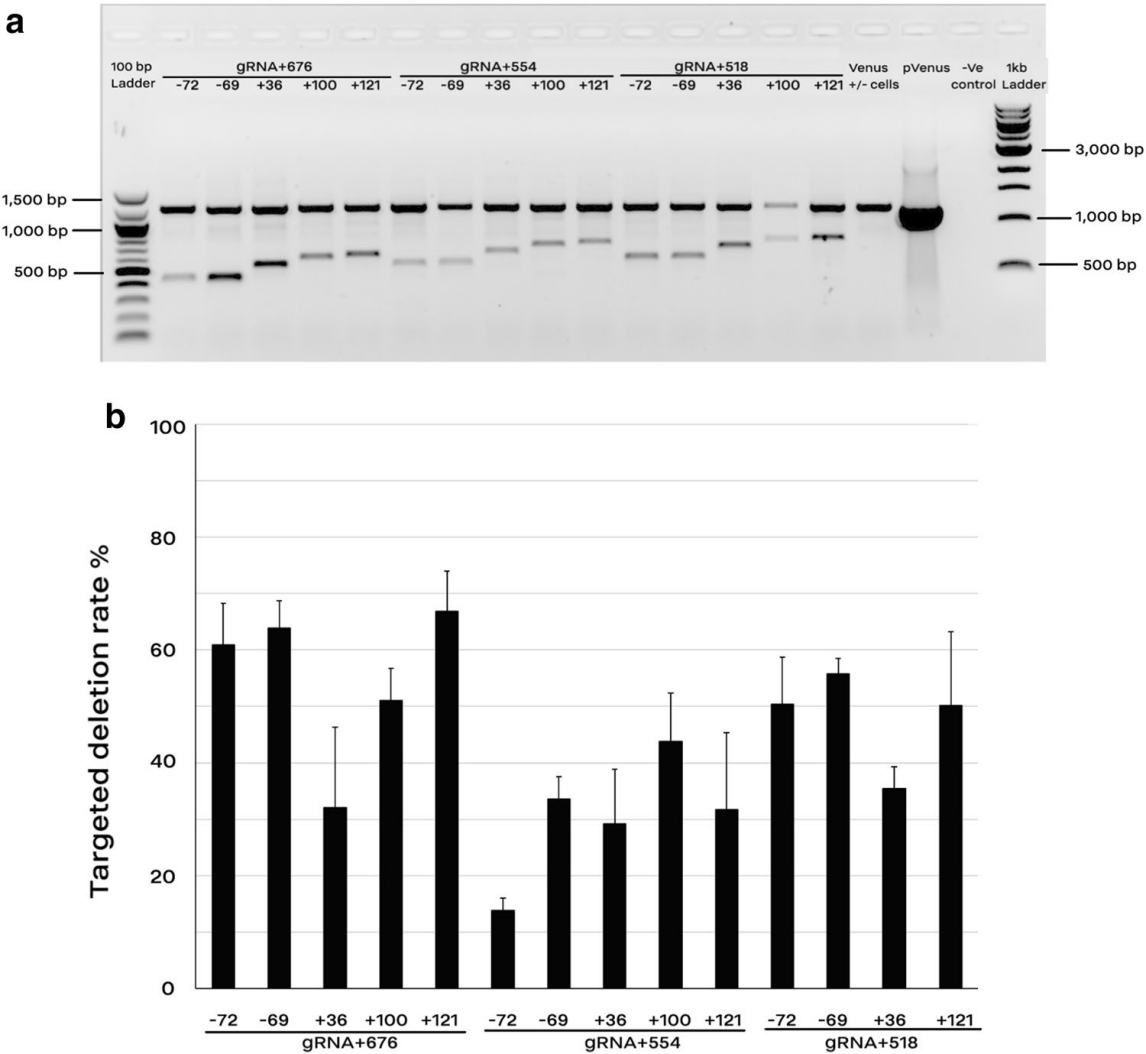
Targeted deletions of Venus transgene by co-electroporation of two gRNA-encoding plasmids. (**a**) End-point PCR showed deletion of 15 different fragments from the original amplicon (1204 bp) using pairwise combination of gRNAs targeting the promoter/ 5′ region (− 72, − 69, + 36, + 100, and + 121) and the 3′ region (+ 518, + 554, and + 676) of Venus cDNA. The primer set (Venus-Forward1 and Venus-Reverse2) amplified both the shortened and original fragments. Genomic DNA from MEF cells carrying no-copy of Venus was considered as the negative controls (− Ve control). (**b**) Digital-PCR results for quantification of the rate of targeted deletion via a primer–probe assay. The primer–probe assay was designed to amplify only the wild type fragment. The deletion rate was calculated as the rate of copy number/µl in the co-electroporated groups divided by that of their control (non-treated) counterparts. Because of a poor DNA quality, the combined group of gRNA + 518 and + 100 was removed from the digital PCR analysis. Data are depicted as average ± standard error (n > 3).

Following making Venus knockouts using indels, we were interested in testing the efficiency of the electrotransfection protocol for making deletions inside of the target gene using two gRNAs. Simultaneously, MEF cells carrying a single-copy of Venus transgene were co-electroporated using 15 combinations of two gRNAs. Co-electroporation of two gRNAs reduced the cell viability significantly (*p* value < 0.05) (Supplementary Figure S6). The result of end-point PCR showed deletions of expected fragments ranging from 398 to 748 bp in the Venus construct using gRNAs pairs (Fig. 5A). Using a flanking primer set (Venus-Forward1 and Venus-Reverse2) both the original transgene and the shortened fragment were detected (Fig. 5A). However, mainly the shortened amplicons were also amplified by another primer set (Venus-Forward1 and Venus-Reverse3) (Supplementary Figure S7). A semi-quantitative PCR approach was carried out to have a rough overview on the deletion rate using different gRNA combinations (Supplementary Figure S8). Quantitative results of the digital PCR clearly showed a significant decrease in the copy number of the transgene in dual gRNAs (Fig. 5B). The deletion rate varied from 13 to 67% using different gRNA combinations.

In overall, the deletion efficiency was more evident using the combinations of gRNA + 676, although gRNA + 554 and + 518 were also efficient for making deletions. Results of the DNA sequencing confirmed the target deletion in co-transfected groups (Supplementary Figure S9).

## Discussion

Nowadays, implementation of the CRISPR/Cas9 technology is a straightforward approach to make genetic engineering in mammalian cells. Electroporation of large plasmids (> 6 kb) is inefficient and associating with a low viability in primary cells^11^. In the current study, we identified OptiMEM-GlutaMAX as the best medium for electroporation of MEF and iPS cells. The electrotransfection efficiencies were quite high throughout the initial setup condition with various pulsing conditions. A > 95% electrotransfection rate was achieved with the optimized pulsing condition. Even the rate of transient expression of reporter genes, as well as the cell viability using the OptiMEM-GlutaMAX in the current study were as high as that of newly reported mechanical–electrical^7^ and nanopore-electrical approaches^12^. A higher rate of cell viability using the OptiMEM-GlutaMAX compared to the Bio-Rad electroporation buffer could partly be due to the difference in their osmolality. However, the osmolality difference did not associate with the electrotransfection efficiency of either iPS or MEF cells. The complete formula of OptiMEM-GlutaMAX medium is not openly released by the producer company. However, it’s a reduced-serum medium that contains insulin and transferrin, as well as trace elements which allow for more than 50% reduction in serum supplementation. Moreover, instead of L-glutamine, it includes the GlutaMAX dipeptide, L-alanine-L-glutamine, which is a stable component in aqueous solutions (https://www.thermofisher.com/de/de/home/life-science/cell-culture/mammalian-cell-culture/media-supplements/glutamax-media.html).

We used plasmids with various sizes, from 6.2 to 13.5 kb, and found a high transfection rate and cell viability irrespective of the plasmid size. It has been evident that electrotransfer of large plasmids (> 6 kb) associates with a high cell toxicity, while the cell transfection rate was also low^11^. However, in our study, electrotransfection of MEF cells using a 13.5 kb was as efficient as a 9.2 kb plasmid, whereas the cell viability remained the same. Therefore the electrotransfection protocol for primary cells in the current study could overcome impediments of hard to transfect cells, as well as high toxicity, and low transfection rate using large plasmids^11^. So far, invention of minicircles, small circular plasmids derived from a normal plasmid but free from all prokaryotic parts of the vector, was considered as one solution to overcome this problem^13^. However, converting a plasmid to a minicircle is time- and cost-consuming and it does not solve the problem of co-transfection of plasmids^11^. Nonetheless, our results showed that electrotransfection of large plasmids is possible with a high efficiency and cell viability in OptiMEM-GlutaMAX medium with no need for a long post-pulse recovery time, as suggested by Lesueur et al.^11^. It has been well-documented that iPS cells are resistant and vulnerable to electroporation^14^. The current study showed that the OptiMEM-GlutaMAX buffer was well suitable for electroporation of murine fibroblasts and iPS cells. It has been suggested that carrying out the electroporation at ice-cold temperature improved the electrotransfection efficiency via increasing the cell viability, and lasting the cell pores for a longer period^15^. However, conducting the electroporation in ice-cold medium did not improve the transfection efficiency in the current study, although the transfection efficiency of ice-cold medium was almost independent of pulsing condition.

Then, we were interested to know if the transient expression is sufficient for genome engineering using the CRISPR/Cas9 technology. Following optimization of the electrotransfection protocol for reporter-encoding transgenes, we implemented the same protocol for transfer of plasmids encoding Cas9 and gRNA aiming to knockout the Venus transgene in MEF cells carrying a single-copy of Venus. The highest rate of Venus knockouts through indels formation (> 95% using gRNA + 100) was as high as the electrotransfection efficiency of reporter-encoding plasmids. The indels rate using plasmids in the current study was higher than reported results by using both plasmid and RNP electrotransfection^16–18^. Following making Venus knockouts using indels, we were interested to test the efficiency of the electrotransfection protocol for making deletions inside of the target gene using double gRNAs. Deletion of expected fragments in the Venus transgene by co-electroporation of two plasmids encoding two different gRNAs was confirmed using endpoint-PCR as well DNA sequencing. Quantification of the deletion efficiency using the digital PCR showed that a 67% targeted deletion was achieved when gRNA + 121 and + 676 were co-electrotransfected. The deletion rate obtained in the current study is higher than previous results using two gRNAs and Cas9 protein^19^ as well as Cas9-encoding plasmids using the BTX electroporation buffer in mouse cell lines^20^.

## Conclusion

In this study, we introduced a highly efficient electrotransfection method for both MEF and iPS cells based on the square-wave pulsing method using OptiMEM-GlutaMAX medium. This plasmid-based delivery method could induce > 95% transfection efficiency for fluorescent reporter genes. Electrotransfection of Cas9/gRNA encoding plasmids resulted in a knockout frequency of up to 98% of a Venus transgene. Apart from indel creation, high rates of targeted deletions were achieved using the co-electroporation of two gRNA-encoding plasmids. Therefore, the developed protocol in the current study can be efficiently used for genetic engineering of mouse primary cells using the CRISPR/Cas9 plasmid system.

## Methods

### The aim and design of the study

In this study, we aimed to develop an efficient electrotransfection method for MEF and iPS cells based on the square-wave pulsing method using a Bio-Rad electroporation device. We optimized the electrotransfection method using two types of reporter plasmids, encoding either Venus or mCherry fluorescent proteins. Then, we assessed the versatility of the developed method for knocking out of the Venus transgene in MEF cells carrying a single-copy of Venus. To approach this aim, we designed and cloned 9 different gRNAs targeting different parts of the Venus transgene. Then, we assessed knockout efficiency of gRNAs by making indels as well as target deletion. For indels production, we conducted electrotransfection of each plasmid individually, while co-electrotransfection of two specific gRNA-encoding plasmids was carried out for the target deletion.

### Materials

All plastic consumables including cell culture flasks and plates, tubes, and filter tips have been purchased from Sarstedt AG & Co. (Germany). Chemical reagents were purchased with the following information: Dulbecco’s phosphate buffered saline without calcium chloride and magnesium chloride (#D6662-10x, Sigma-Aldrich, Germany); Opti-MEM 1x + GlutaMAX, reduced serum medium (#1854076, Gibco, Life Technologies, Germany); DMEM high glucose (4.5 g/l) w/o L-glutamine (#DMEM-HXA, Capricorn Scientific GmbH, Germany); OptiMEM + Glutamine (#1985, Gibco, Life Technologies, Germany), L-glutamine for cell culture (#A3704,0100, Applichem, Germany); MEM nonessential amino acids solution (100x) (#NEAA-B, Capricorn Scientific GmbH, Germany); 2-mercaptoethanol (#BCBS5481, Sigma-Aldrich, Germany); penicillin/streptomycin solution (100x) (#PS-B, Capricorn Scientific GmbH, Germany); trypsin–EDTA (10x) (#L11-003, GE Healthcare, PPA Laboratories GmbH, Austria); fetal bovine serum (#10270–106, Gibco, Thermo Fisher Scientific, Germany); dimethyl sulfoxide (#D4540-500ML, Sigma-Aldrich, Germany); LIF (hBA-FL) (#sc-4377, Santa Cruz Biotechnology, Germany); sodium pyruvate (#P2256, Sigma-Aldrich, Germany); gelatin from bovine skin (#G939-100G, Sigma-Aldrich, Germany); and Hoechst 33342 (#62249, Thermo Scientific, Germany). For electroporation, a Gene Pulser Xcell system with CE Module from BioRad (Germany) was used with 4 mm electroporation cuvettes (#748052, Biozym, Germany).

### Plasmids

The *Sleeping Beauty* transposase was encoded in pCMV-T7_SB100X (4756 bp) which contains the hyperactive variant 100 of SB under the CMV promoter^21^. Two reporter plasmids, pT2-Venus (6301 bp) encoding the Venus fluorescent marker under CAGGS promoter and pT2-mCherry (7756 bp) encoding mCherry, were used^21^. In addition, a 13.5 kb plasmid, pSGD-Lys-72, was also used as a large plasmid which encodes human lysozyme under CAGGS promoter followed by Cas9 protein which was separated from puromycin resistance by a T2A peptide and expressed under CMV promoter, and gRNA-72 under U6 promoter. All pT2-Venus, pT2-mCherry, and pSGD-Lys-72 plasmids contained inverted terminal repeats (ITRs) of the SB transposase. The piggyBac (PB) reprogramming transposon involved a CAGGS promoter driven cassette containing Oct4, Sox2, Klf4, and c-Myc, which were separated by sequences encoding the self-cleaving 2A peptides, and flanked by PB-ITRs^22^.

For the CRISPR/Cas9 study, we modified pX459 plasmid (9151 bp) which encodes a Cas9 protein followed by puromycin resistance under CAGGS promoter/enhancer, as well as the gRNA scaffold under the U6 promoter. All gRNAs were synthetized and cloned into the backbone vector based on the Franham protocol^23^. Briefly, gRNAs were selected to target Venus promoter or open reading frame (ORF) via CRISPOR software, available online (https://crispor.tefor.net/)^24^. The list of gRNAs is available in Supplementary Table S1. The 100 µM forward and reverse oligos were annealed in 10 µl reaction volume by incubation in a water bath containing pre-boiled water and letting it to cool down to room temperature. The pX459 plasmid was digested with *Bbs*I-HF (NEB #R3539) at 37 °C for 10 min followed by gel purification using NucleoSpin Gel and PCR Clean-up Midi kit (#740,986.20, Machery/Nagel). The purified fragment was kept at − 20 °C for further applications. Ligation of the annealed oligo-duplex with the digested pX459 was carried out as follow: diluted oligo-duplex (1:20 ratio from the 10 µM source) (1 µl), digested pX459 vector (50 ng), 10 × T4 DNA ligase buffer (2 µl), and T4 DNA ligase (1 µl) in a 20 µl final reaction. The ligation reaction was incubated at room temperature for 1 h. Transfection of the ligation mixture was carried out into NEB 5-alpha Competent *E. coli* (# C2987I) following incubation of 10 µl of the ligation reaction with the thawed competent cells on ice for 20 min and then at 37 °C for 5 min. Then, 400 µl of SOC medium was added into the transformation tube, incubated at 37° C for 30 min, plated on agar plates supplemented with 100 µg/ml ampicillin, and incubated at 37 °C overnight. From the cultured plate, 10 colonies were picked and each was cultured in 3 ml LB medium followed by miniprep plasmid extraction (Genejet Plasmid miniPrep kit, #K0502). The extracted plasmids were simultaneously digested by *Bbs*I and *Eco*RV restriction enzymes (Fermentas). Plasmids with the expected bands of 6244 and 2928 bp were sent for sequencing using the gRNA-Sequencing primer (Supplementary Table S2). Alignment of the sequenced DNA with the Venus sequence was carried out using the Geneious (version 11.1.5) software.

### Cell culture

A 1% gelatin solution was prepared by stirring 250 ml PBS and 2.5 g gelatin for one hour without heating, and the solution was then autoclaved. Five milliliters of the 1% gelatin was poured into a 6-well plate and aspirated after 1 min. Plates were left to be dried under the laminar flow hood for 30 min.

### **Cell lines**

All cell lines were derived either during the current project or from previous projects in this lab. We used mouse embryonic fibroblast cells (MEFs), carrying either no- or a single-copy of the Venus transgene (Venus ±)^25^. Mouse embryonic fibroblast cells were isolated from day 11 embryos of the wild type or Venus ± embryos . Embryos were eviscerated and sliced, and small tissue pieces were cultivated with 5 ml fibroblast medium (DMEM with 10% FBS, 2 mM L-glutamine, 1% penicillin–streptomycin, 1% nonessential amino acids, and 0.01% β-mercaptoethanol) in T75 flasks. After 3 days of the incubation, propagated cells were trypsinized, passaged, and used for cell culture and transfection. Moreover, mouse iPS cells were derived from MEF cell lines^22^. Briefly, MEFs were electroporated with plasmids containing a 4-factor reprogramming *PiggyBac* (PB) transposon and the helper plasmid containing a hyperactive PB transposase^22^. Electroporated cells were cultured in gelatinized 6-well plates and iPS medium. Presumptive iPS colonies were picked under microscope, and plated into individual wells of 96-well plates containing trypsin. Trypsin was neutralized with DMEM and 10% FCS, and the cells suspension was dispensed into individual wells of gelatinized 96-well plates 22.

### Cell electroporation

To assess the transfection efficiency using reporter plasmids, MEFs and iPS cells without the Venus reporter were used. Both MEFs and iPS cells were cultured in 6-well plates. From subconfluent cultures, cells from each well of a 6-well plate were used for each electroporation reaction using both iPS and MEFs cells. Cells were washed once with 2 ml PBS and then trypsinized with 200 µl trypsin–EDTA. Trypsin activity was inhibited by 4 ml PBS per well. Cells were centrifugation at 1000 rpm for 3 min, and the cell pellet was resuspended into 250 µl of the electroporation medium. We compared electroporation efficiency of three buffers/media, including PBS, OptiMEM-GlutaMAX, and the Bio-Rad electroporation buffer with osmolality of 0.275, 0.290, and 0.430 (Osmol/kg)^26^, respectively. In an initial experiment, the highest transfection efficiency of iPS cells were reached by OptiMEM-GlutaMAX medium (Supplementary Figure S1). Pulse voltage was constrained to 250 V for iPS cells, so that a 70–80% cell viability was achieved. Then, the effect of pulse number and duration as well as medium temperature was optimized for iPS cells (Figures S2). Finally, we reached the following optimized condition for electroporation of mouse iPS cells. Twenty micrograms of plasmid DNA were mixed with cells in a volume of 250 µl, and the cell-plasmid mixture was transferred into the 4 mm cuvettes, and underwent the following electroporation program: The square-wave protocol with 250 V, each 10 ms pulse length, 2 pulses, and 10 s pulse interval. We used 20 µg plasmid DNA (1.5–2.5 µg/µl) in each electroporation reaction. MEFs underwent the same electroporation program which was optimized for iPS cells, except an increase of the plus voltage to 300 V, so that a 70–80% cell viability was also attained. After the electroporation process, cells were transferred into the culture medium in 6-well plates and incubated at 37 °C and 5% CO_2_.

### Venus knockout using one and two gRNAs

For the CRISPR/Cas9 experiment, we used MEFs cells with a single-copy of Venus for making KO-Venus cells^25^. The above-mentioned optimized protocol for cell electrotransfection was also used for Cas9/gRNA encoding plasmids. Knockout of *Venus* was carried out either by indels using 20 µg (1.5–2.5 µg/µl) of one modified pX459 plasmid or by deletions via co-electroporation of two modified pX459 plasmids (each 20 µg per reaction). To consider the deletion efficiency of various sizes, three gRNAs which target the 3′-region of Venus transgene (gRNA + 518, + 554, and + 676) were pair-wisely co-electroporated with five gRNAs which targeted either the promoter or the 5′-region of Venus (gRNA − 72, − 69, + 36, + 100, and + 121).

### DNA extraction and end-point PCR

DNA was extracted from each using the proteinase K and salting-out method^27^. Briefly, confluent cells in 6-well plate were trypsinized with 250 µl trypsin–EDTA, directly transferred into a 1.5 ml tube containing 1 ml of cell lysis buffer, which included 0.2 mg/ml proteinase K, 150 mM NaCl, 10 mM Tris, 10 mM EDTA, and 0.1% sodium dodecyl sulfate (SDS), and were incubated overnight at 55 °C in a shaker-incubator. Then, 500 µl saturated NaCl was added into each tube, converted for 5–10 times, and centrifuged with high speed at room temperature. Supernatants were transferred into two new tubes and underwent ethanol precipitation with absolute ethanol followed by washing with 70% ethanol using centrifugation with high speed at 4 °C. The DNA pellet was solved in 200 µl distilled water and underwent an extra round of ethanol precipitation. Extracted genomic DNA was quantified by a spectrophotometer (Nanodrop, USA), and kept at − 20 °C till further usage. Different primer pairs which cover the Venus transgene were designed using the Primer3 webtool, available in the NCBI website (Supplementary Table S2). The PCR reaction comprised of 50 ng genomic DNA, 5X PCR buffer, 2.5 mM MgCl2, 5 × PCR buffer, 10 µM forward/ reverse primer, 0.2 mM dNTPs, and 1 I.U. GoTaq DNA polymerase (#9PIM300, Promega, Germany). The end-point PCR was carried out by Bio-Rad T100 thermal cycler with the following program: 94 °C for 2 min, and 34 cycles of 94 °C for 45 s, 61 °C for 30 s, and 72 °C for 45 s, followed by a 5-min incubation at 72 °C. PCR products were loaded on a 0.7% agarose gel, and the banding pattern was visualized. DNA was sequenced using the sanger method (LGC Genomics, Germany). The indels spectrum was analyzed using the TIDE (Tracking of Indels by Decomposition) webtool (https://shinyapps.datacurators.nl/tide/).

### Detection of target deletion using the digital PCR

A primer–probe assay-1 was designed to target a common segment in the targeted deletion of all 15 combination of dual gRNAs using the IDT online software (Supplementary Table S2). The Prime Time 5′ 6-FAM/ZEN/3′ IBFQ probe was synthetized by IDT (Integrated DNA Technologies, USA). The primer pair were separately ordered from Eurofins Genomics (Germany). We also used the 2X QuantStudio 3D Digital PCR Master Mix V2 (#A26358, Thermo Fisher Scientific, USA), and QuantStudio 3D Digital PCR 20 K Chip Kit v2 (#A26316, Thermo Fisher Scientific, USA). Each primer–probe mixture (20x) was produced to include 5 mM probe and 18 mM each primer, which gave us final concentrations of 250 and 900 nM for the probe and each primer, respectively. The digital PCR reaction involved 8.7 µl of 2x master mix, 0.87 µl of 20x primer–probe assay, 1.83 µl distilled water, and 6 µl of DNA. First, the PCR reaction was optimized by using a serial dilution of genomic DNA concentration. Genomic DNA extracted from MEF cells carrying a single-copy of Venus was digested by *Hae*III restriction enzyme at 37 °C for 3 h. The digestion reaction was inactivated by 10 min incubation at 65 °C. The digital PCR was optimized by using 20, 40, and 80 ng/µl of the digested genomic DNA. A 16 µl fraction of the PCR reaction was properly pipetted and dispensed on the chip using the QuantStudio 3D Digital PCR Chip Loader. The chip was filled by the immersion fluid provided by the Chip kit, and sealed firmly with the chip sealant. Digital PCR was carried out by the ProFlex 2X Flat PCR System with the following program: 96 °C for 10 min, followed by 39 cycles of 60 °C for 2 min and 98 °C for 30 s, as well as one extra step of incubation at 60 °C for 2 min, and ended with storing at 10 °C. The cover temperature was 70 °C, as suggested by the instructor. Samples were kept at ambient temperature for 3 h following the PCR reaction, and then used for imaging using the QuantStudio 3D Digital PCR Instrument. The emission of FAM dye was recorded and analyzed by the instrument and the data was further analyzed by the online software of QuantStudio 3D AnalysisSuite (Thermo Fisher Scientific). Based on the optimized DNA concentration, all genomic DNAs were digested by *Hae*III restriction enzyme and diluted to 40 ng/µl for using in the PCR reaction. The number of Venus copies/µl of the PCR reaction in each sample was divided by that of the positive control group (40 ng/µl of MEF cells carrying a single-copy of the Venus transgene). It should be mentioned that the quality of genomic DNA was also confirmed by a semi-quantitative PCR, including 94 °C for 4 min, and 32 cycles of 94 °C for 45 s, 60 °C for 30 s, and 72 °C for 20 s, followed by a 5-min incubation at 72 °C (Supplementary Figure S8). Except for the combined group of gRNA + 518 and gRNA + 100, results of semi-quantitative PCR were consistent for amplification of the housekeeping gene in all groups. Therefore, this group was excluded for quantification of the target deletion rate.

### Fluorescence microscope imaging and flow cytometry analysis

For the fluorescent imaging, cells in each well of a 6-well plate were washed once with PBS, the medium was replaced with the transparent OptiMEM (without phenol) supplemented with Hoechst 33342 (2: 10,000 ratio from 1 mg/ml stock solution), and incubated at 37 °C for 30 min. Transfection efficiency for mCherry and Venus reporters were assessed either by FACS machine or fluorescent microscopy. Cell viability was defined as cell numbers in the electroporated group divided by the cell number in the control group which underwent no electroporation. Knockout efficiency of various gRNA sequences targeting the Venus reporter was assessed 10 days after the electroporation. A flow cytometer, MACSQuant Analyzer, was also used to assess cell transfection rate. We used Blue 488 nm in B1 channel with 525/550 nm filter and Yellow 461 nm Y2 channel with 615/20 nm filter for detection of Venus and mCherry proteins, respectively.

### Statistical analysis

Means comparison was carried out using the least significant difference (LSD) test (p-values 0.05). All experiments were carried out at least three times.

### Ethics approval and consent to participate

The German legislation (Tierschutz-Versuchstierverordnung-TierSchVerV §14) does not require an ethics approval for humanely killing of laboratory animals for the purpose of organ removal.

## Supplementary information

Supplementary Information.

## Data Availability

The datasets used and/or analyzed during the current study are available from the corresponding author.

## Abbreviations

CRISPR: Clustered regularly interspaced short palindromic repeats
Cas9: CRISPR-associated protein 9
gRNA: Guide RNA
iPS: Induced pluripotent stem cell
KO: Knockout
MEF: Mouse embryonic fibroblast
RNPs: Ribonucleoprotein

## References

[1] Sakuma T, Nishikawa A, Kume S, Chayama K, Yamamoto T (2014). Multiplex genome engineering in human cells using all-in-one CRISPR/Cas9 vector system. Sci. Rep..

[2] Han X (2015). CRISPR-Cas9 delivery to hard-to-transfect cells via membrane deformation. Sci. Adv..

[3] Liu C, Zhang L, Liu H, Cheng K (2017). Delivery strategies of the CRISPR-Cas9 gene-editing system for therapeutic applications. J. Control. Release.

[4] Gresch O (2004). New non-viral method for gene transfer into primary cells. Methods.

[5] Weaver JC, Smith KC, Esser AT, Son RS, Gowrishankar T (2012). A brief overview of electroporation pulse strength–duration space: A region where additional intracellular effects are expected. Bioelectrochemistry.

[6] Neumann E, Schaefer-Ridder M, Wang Y, Hofschneider P (1982). Gene transfer into mouse lyoma cells by electroporation in high electric fields. The EMBO journal.

[7] Ding X (2017). High-throughput nuclear delivery and rapid expression of DNA via mechanical and electrical cell-membrane disruption. Nat. Biomed. Eng..

[8] Lakshmipathy U (2004). Efficient transfection of embryonic and adult stem cells. Stem cells.

[9] Yang Z, Chang L, Chiang C-L, James Lee L (2015). Micro-/nano-electroporation for active gene delivery. Curr. Pharm. Des..

[10] Kaneko T, Sakuma T, Yamamoto T, Mashimo T (2014). Simple knockout by electroporation of engineered endonucleases into intact rat embryos. Sci. Rep..

[11] Lesueur LL, Mir LM, André FM (2016). Overcoming the specific toxicity of large plasmids electrotransfer in primary cells in vitro. Mol. Ther. Nucleic Acids.

[12] Cao Y (2019). Nontoxic nanopore electroporation for effective intracellular delivery of biological macromolecules. Proc. Natl. Acad. Sci..

[13] Joubert V, André FM, Schmeer M, Schleef M, Mir LM (2013). Increased efficiency of minicircles versus plasmids under gene electrotransfer suboptimal conditions: an influence of the extracellular matrix. Minicircle Miniplasmid DNA Vectors Future Nonviral Viral Gene Transfer.

[14] Qian K (2014). A simple and efficient system for regulating gene expression in human pluripotent stem cells and derivatives. Stem Cells.

[15] Potter H, Heller R (2017). Transfection by electroporation. Curr. Protoc. Immunol..

[16] Liang X (2015). Rapid and highly efficient mammalian cell engineering via Cas9 protein transfection. J. Biotechnol..

[17] Jacobi AM (2017). Simplified CRISPR tools for efficient genome editing and streamlined protocols for their delivery into mammalian cells and mouse zygotes. Methods.

[18] Liang X, Potter J, Kumar S, Ravinder N, Chesnut JD (2017). Enhanced CRISPR/Cas9-mediated precise genome editing by improved design and delivery of gRNA, Cas9 nuclease, and donor DNA. J. Biotechnol..

[19] Kraft K (2015). Deletions, inversions, duplications: engineering of structural variants using CRISPR/Cas in mice. Cell Rep..

[20] Canver MC (2017). Characterization of genomic deletion efficiency mediated by clustered regularly interspaced short palindromic repeats (CRISPR)/Cas9 nuclease system in mammalian cells. J. Biol. Chem..

[21] Ivics Z (2014). Germline transgenesis in rodents by pronuclear microinjection of Sleeping Beauty transposons. Nat. Protoc..

[22] Talluri TR (2014). Non-viral reprogramming of fibroblasts into induced pluripotent stem cells by Sleeping Beauty and piggyBac transposons. Biochem. Biophys. Res. Commun..

[23] Guo Y (2018). CRISPR-mediated deletion of prostate cancer risk-associated CTCF loop anchors identifies repressive chromatin loops. Genome Biol..

[24] Haeussler M (2016). Evaluation of off-target and on-target scoring algorithms and integration into the guide RNA selection tool CRISPOR. Genome Biol..

[25] Garrels W (2016). Cytoplasmic injection of murine zygotes with Sleeping Beauty transposon plasmids and minicircles results in the efficient generation of germline transgenic mice. Biotechnol. J..

[26] Hyder I, Eghbalsaied S, Kues WA (2020). Systematic optimization of square-wave electroporation conditions for bovine primary fibroblasts. BMC Mol. Cell Biol..

[27] Minematsu T, Sugiyama M, Tohma Y, Tajima A, Kanai Y (2004). Simplified DNA extraction methods for sexing chick embryos. J. Poult. Sci..

