# Supplementary material for: A versatile bulk electrotransfection protocol for murine embryonic fibroblasts and iPS cells
Source: Sci Rep. 2020 Aug 7;10:13332. doi: 10.1038/s41598-020-70258-w (PMC7414887; doi:10.1038/s41598-020-70258-w)
Supplement: Supplementary file 1 — Supplementary Information. [file 41598_2020_70258_MOESM1_ESM.docx]

**Supplementary Materials**

**A versatile bulk electrotransfection protocol for murine embryonic fibroblasts and iPS cells**

Shahin Eghbalsaied^1,2^, Iqbal Hyder^1^, Wilfried A. Kues^1*^

^1^Department of Biotechnology, Friedrich-Loeffler-Institut (FLI), Neustadt, Germany

^2^Department of Animal Science, Isfahan (Khorasgan) branch, Islamic Azad University, Iran.

**^*^Corresponding author:** [wilfried.kues@fli.de](mailto:wilfried.kues@fli.de)

Friedrich-Loeffler-Institut (FLI), Institute of Animal Health, Neustadt, Germany.
**Postal address:** Höltystr. 10, 31535 Neustadt, Germany.

**Telephone:** +49 5034 871 5120
**Fax:** +49 5034 871 5101

**Table S1.** The sequence of gRNAs specific for Venus transgene. These gRNAs were designed by CRISPOR online software.

| Name | sequence (5′-3′) |
| --- | --- |
| gRNA-252 | CTTCGCCCGCGCCCGCTAGA |
| gRNA -72 | TTCGGCTTCTGGCGTGTGAC |
| gRNA -69 | GGCTTCTGGCGTGTGACCGG |
| gRNA +36 | CGAGGAGCTGTTCACCGGCG |
| gRNA +100 | AAGTTCTCCGTGAGCGGCGA |
| gRNA +121 | GGCGAGGGCGACGCCACCTA |
| gRNA +518 | AGGCACAACATCGAGGACGG |
| gRNA +554 | AGCACGGGGCCGTCGCCGAT |
| gRNA +676 | CTGGAGTTCGTGACCGCCGC |

**Table S2.** Primers and probes for amplification and detection of Venus transgene

| Primer/probe Name | Sequence (5′-3′) | Tm | Amplicon length (bp) |
| --- | --- | --- | --- |
| Venus-Forward1  Venus-Reverse1 | ACGCGTTAAGATACATTGATGAGTT  AGGACGACGGCAACTACAAG | 59.4  60.0 | 556 |
| Venus-Forward2  Venus-Reverse2 | CTTGTAGTTGCCGTCGTCCT TGCCTTTTATGGTAATCGTGCG | 60.0  59.6 | 668 |
| Venus-Forward1  Venus-Reverse2 | ACGCGTTAAGATACATTGATGAGTT  TGCCTTTTATGGTAATCGTGCG | 59.4  59.6 | 1,204 |
| Venus-Forward1  Venus-Reverse3 | ACGCGTTAAGATACATTGATGAGTT  TTGCCTTTTATGGTAATCGTGCG | 59.4  59.8 | 1,205 |
| HPRT-Forward  HPRT-Reverse | CTCCTCAGACCGCTTTTTGC  TAATCACGACGCTGGGACTG | 60.5  60.5 | 75 |
| Assay-Forward  Assay-Reverse  Probe | CCCTTCAGCTCGATTCTGTT  GCAGGAGAGAACCATCTTCTT  56-FAM-CCTCGGCTC-Zen-TGGTCCTGTAGTTGC-3IABkFQ | 61.9  61.7  68.0 | 102 |
|  |  |  | - |
| gRNA-Sequencing | GGACTATCATATGCTTACCGTAAC | 62.0 | - |


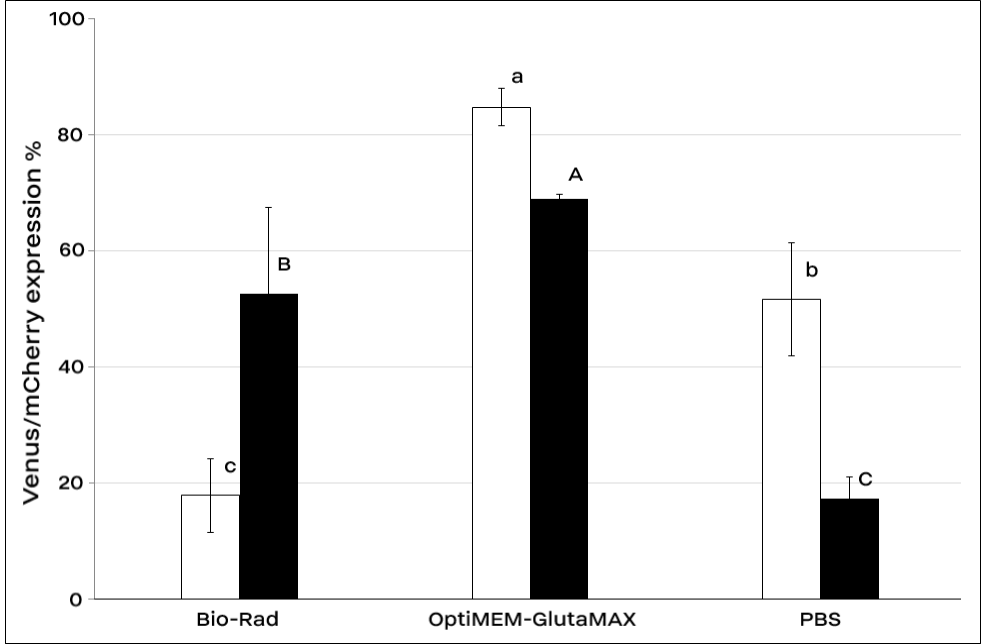


**Figure S1. Media effect on the electrotransfection efficiency of mouse iPS cells.** Three media (Bio-Rad buffer, OptiMEM-GlutaMAX, and PBS) were compared. In each electroporation reaction, 20 µg of either pT2-Venus or pT2-mCherry was pre-mixed with cells and underwent electroporation using the square-wave protocol with 200 V voltage, 10 ms pulse length, and 4 mm cuvette. The expression of the fluorescent reporter was assessed 36 h after electroporation under a fluorescence microscope. White and black bars are electrotransfection efficiency and cell viability, respectively. Bars with different a, b, c or A, B, C letters are significantly different (p-value < 0.05). Results are means and standard deviation (n > 3).


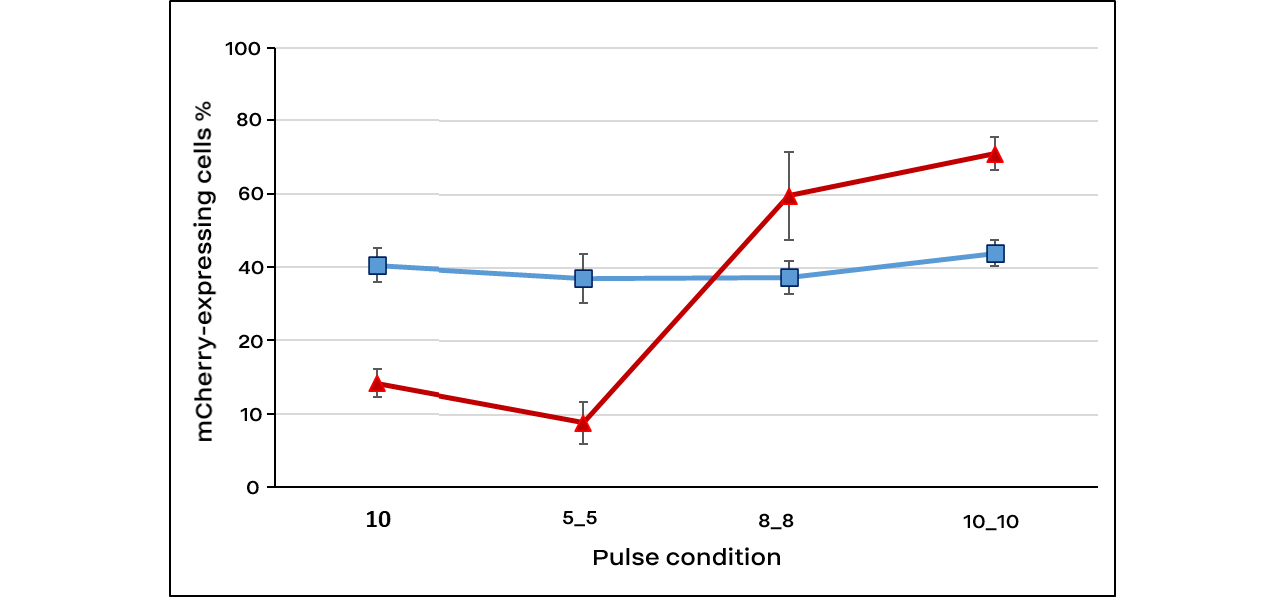


**Figure S2. The effect of temperature and pulsing condition on electroporation efficiency of mouse iPS cells**. Twenty microgram of a plasmid carrying mCherry transgene under CAGGS promoter was used for the electrotransfection. A square-wave protocol of 250 V, either single pulse for 10 ms or double pulses each for 5, 8, or 10 ms with 10 s pulse interval in 4 mm cuvettes, and 250 µl OptiMEM-GlutMAX was applied. Cuvettes were kept either on ice for 15 min (blue squares) or at room temperature (red triangles) before and after the pulsing conduction (n > 3).


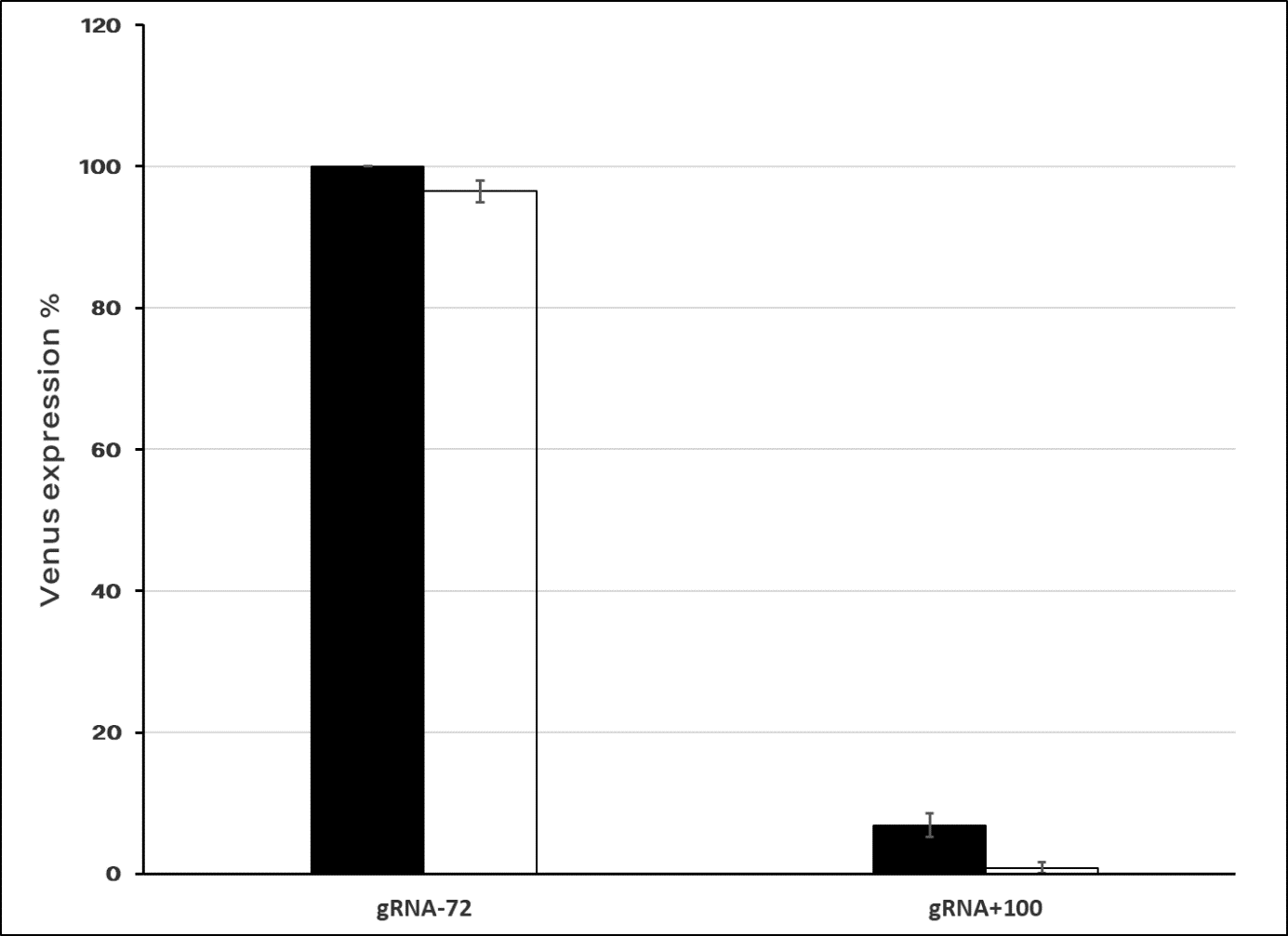


**Figure S3. Knockout efficiency of Venus transgene in MEF cells treated with or without puromycin.** Two plasmids encoding the Cas9 protein and a gRNA, gRNA-72 targeted the promoter and gRNA+100 targeted the coding region of the Venus transgene, were electroporated into MEF cells carrying a single-copy of Venus using the optimized protocol. Cells were treated one-day post-transfection either with (white bars) or without puromycin (black bars). Cells were screened for the Venus expression under a fluorescence microscope 10 days after the electroporation (n > 3).


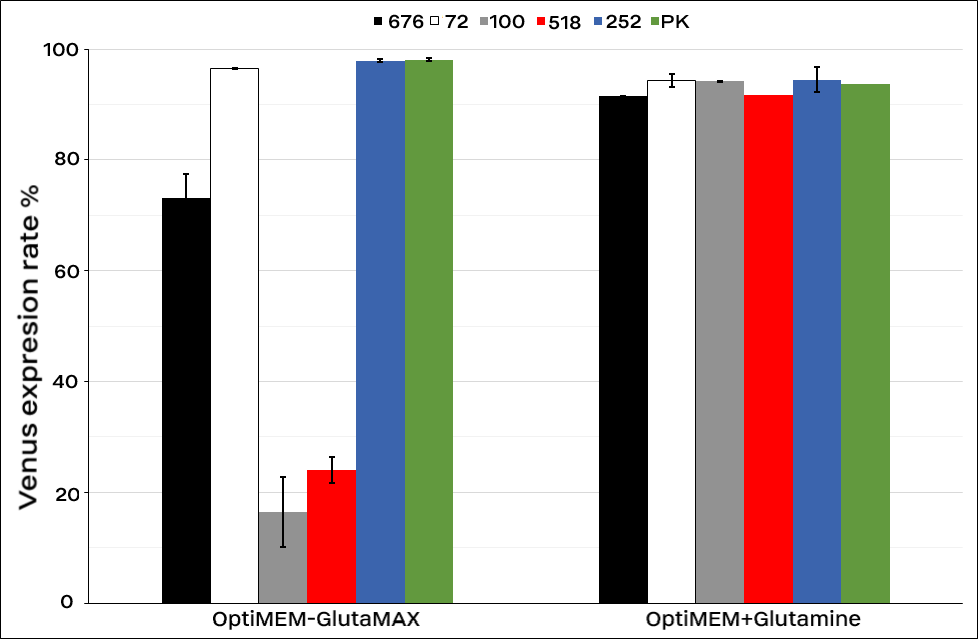


**Figure S4.** **Comparison of OptiMEM-GlutaMAX vs. standard OptiMEM (+Glutamine).** MEF cells carrying a single-copy of Venus were electrotransfected using the modified pX459 plasmid, encoding gRNA+100, +518, and +676. The electroporation medium was 250 µl of either OptiMEM-GlutaMAX (#1854076, Gibco, Germany) or OptiMEM+Glutamine (#31985, Gibco, Germany). Cells were screened for the Venus transgene under a fluorescence microscope 10 days after the electroporation (n > 3). Cells with one copy of Venus was considered as the positive control (PK) for Venus expression.


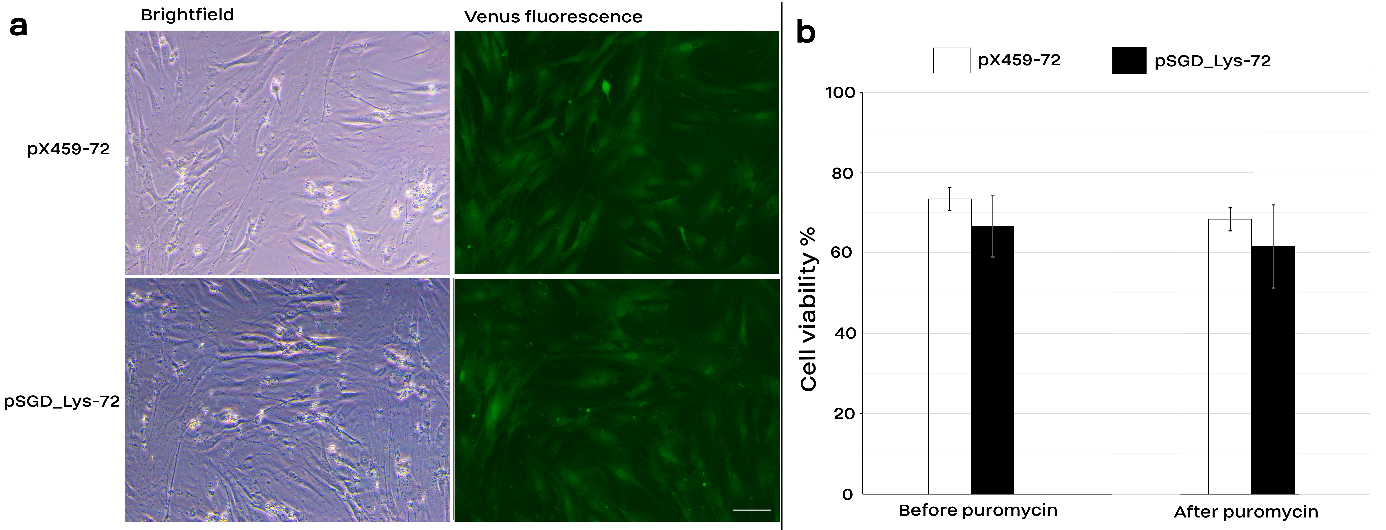


**Figure S5. Viability of electrotransfected cells using large plasmids.** Large plasmids ranging from 9.2 kb (pX459-72) to 13.5 kb (pSGD-Lys-72) were used for electrotransfection of MEF cells carrying a single-copy of Venus. Cell viability was measured 24 h after the elctrotransfection (before the puromycin selection) and 24 h after the puromycin selection. Following 24 h after the electroporation process, cells underwent a one-day puromycin selection. **a**) Results of fluorescent microscopy of cells after 24 h post-puromycin selection. **b**) Cell viability 24 h after the electroporation (before puromycin treatment) and 24 h after the puromycin selection (n > 3). Cell viability was defined as cell numbers in the electroporated group divided by the cell number in the control group which underwent no electroporation. Scale bar equals 10 µm.


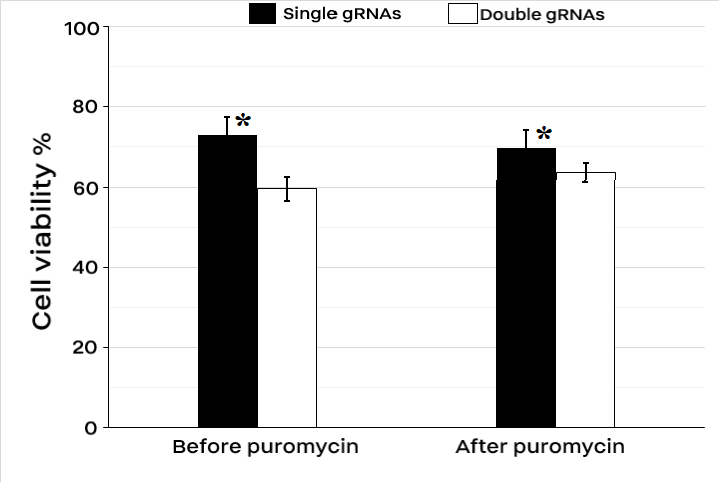


**Figure S6. Cell viability after the electrotransfection suing the single- and double-gRNAs.** MEF cells carrying a single-copy of Venus were electrotransfected using either of the modified pX459 plasmid, encoding gRNA-252, -72, -69, +36, +100, +121, +518, +554, and +676. This group was considered as single-gRNAs. Pairwise combinations of gRNAs targeted the promoter or 3´- region (-72, -69, +36, +100, and +121) and the 5´-region (+518, +554, and +676) of Venus were co-electroporated. Cell viability was defined as cell numbers in the electroporated group divided by the cell number in the control which underwent no electroporation. Cell viability was measured 24 h after the elctrotransfection (before the puromycin selection) and 24 h after the puromycin selection. Results of all single-gRNAs and all double-gRNAs were pooled and used for mean comparison (n > 3). Asterisk (*) indicates a statistically significant difference (p-value < 0.05).


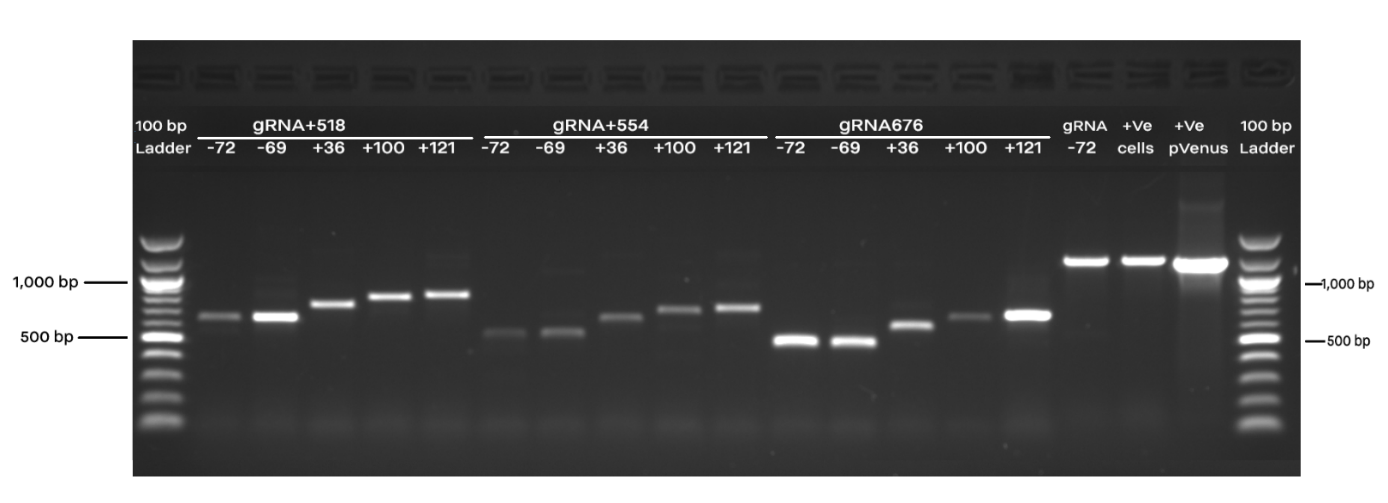


**Figure S7. Targeted deletions in Venus transgene by co-electroporation of two gRNA-encoding plasmids.** End-point PCR using primer Venus-Forward1 and Venus-Reverse3 showed deletion of five different fragments from the original amplicon (1,205 bp) using pairwise combination of gRNAs targeting the promoter or 5´ regions (-72, -69, +36, +100, and +121) and 3´ region (+518, +554, and +676) of Venus. The shortened fragments have mainly been amplified using this primer set in the co-electrotransfected groups. As control groups, we used genomic DNA from MEF cells carrying a single-copy of Venus (+Ve cells) and pVenus plasmid (+Ve pVenus).


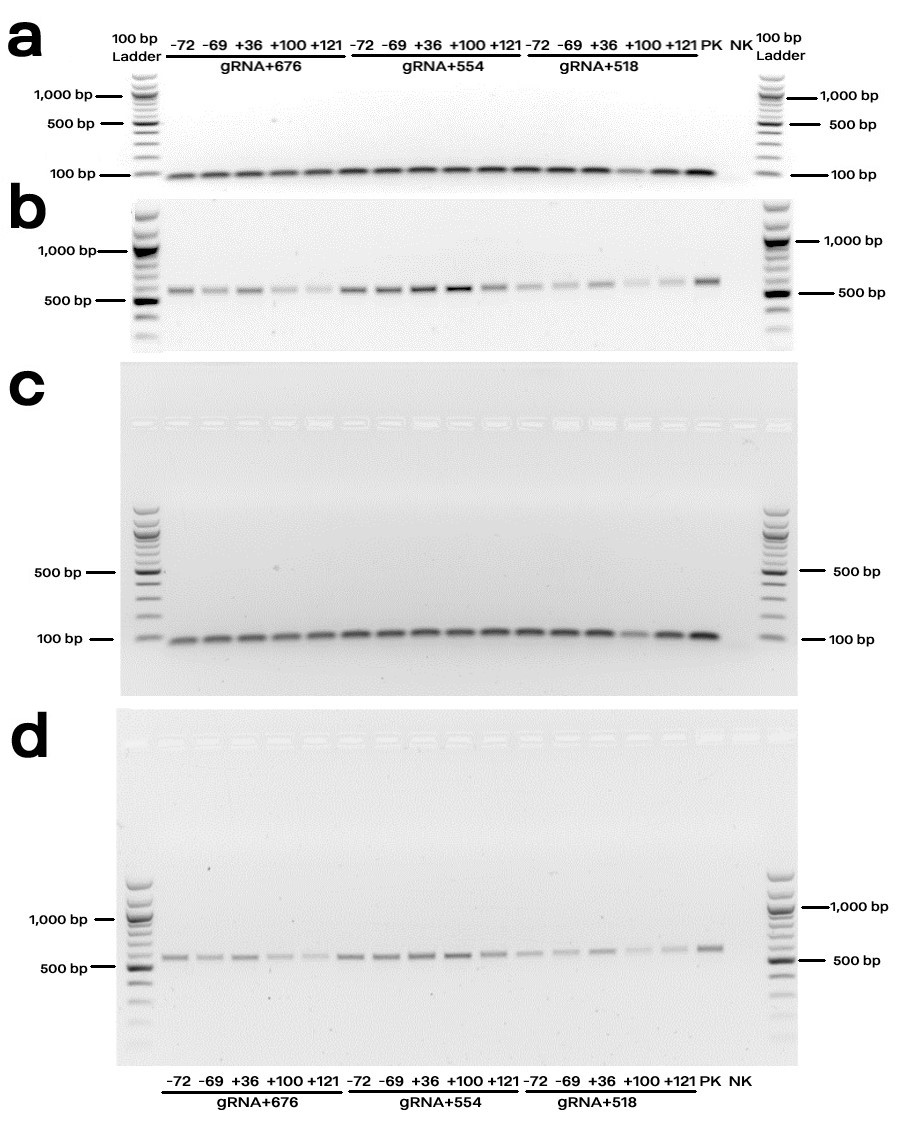


**Figure S8. Semi-quantitative PCR for assessment of targeted deletions in Venus transgene.** Pairwise combinations of gRNAs targeted the promoter or 3´- region (-72, -69, +36, +100, and +121) and the 5´-region (+518, +554, and +676) of Venus was carried out to perform target deletion in the Venus transgene. A semi-quantitative PCR with 32 cycles was carried out using primer pairs which were specific for the targeted deletion regions of all 15 combination types in the Venus transgene. **a**) Amplification of a 75 bp fragment in the HPRT as an endogenous gene. **b**) Amplification of a 556 bp fragment using Venus-Forward1 and Venus-Reverse1 primers in the Venus transgene. The reverse primer was located in the common deleted region, so only the wild type sequence with no target deletion was amplified. **c** and **d** are original images from section **a** and **b**, respectively. Genomic DNA from MEF cells carrying a single-copy and no-copy of Venus was considered as the positive (PK) and negative controls (NK), respectively.

**
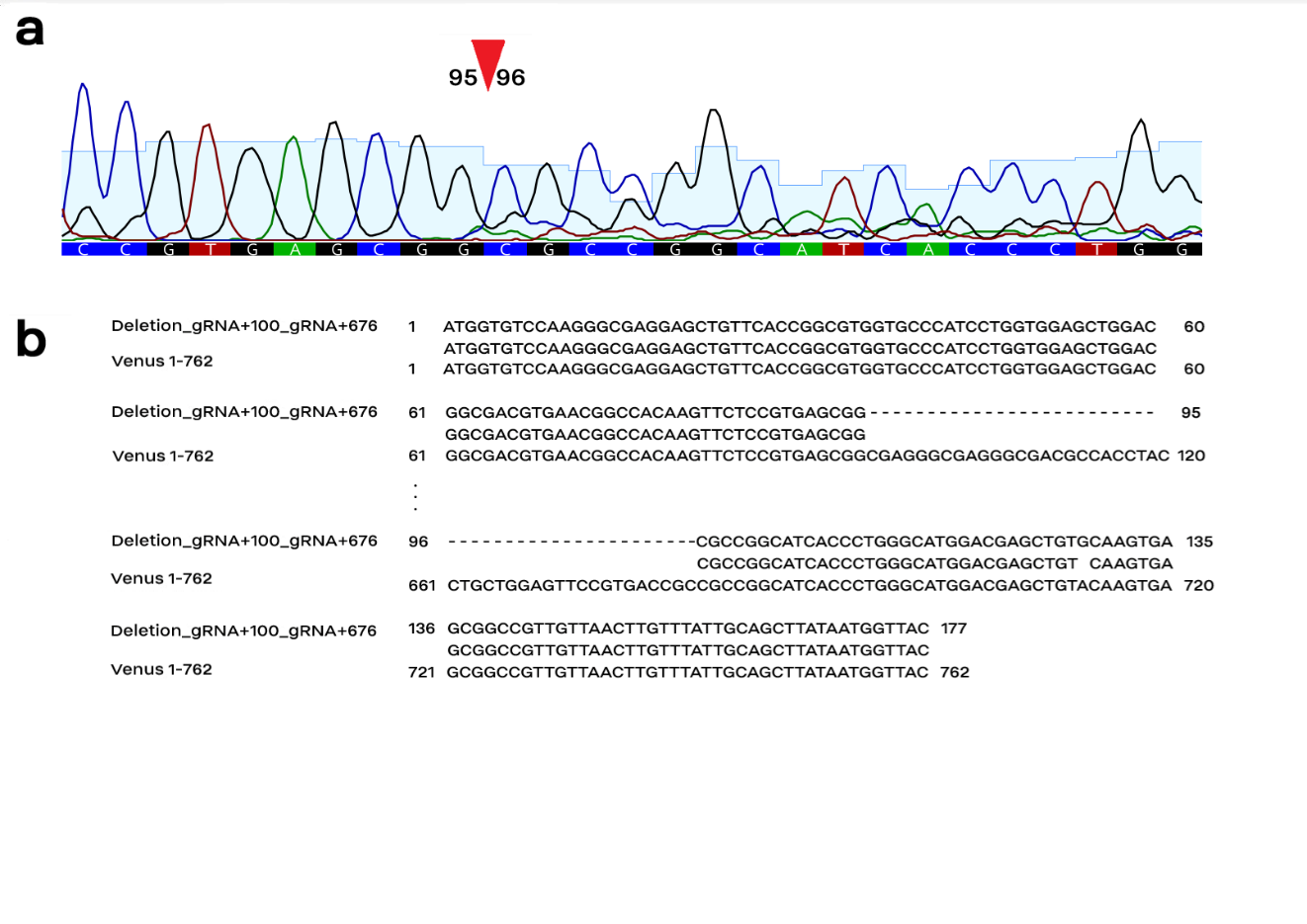
**

**Figure S9. Sequencing results of Venus transgene which underwent a targeted deletion.** MEF cells carrying a single-copy of Venus were co-electrotransfected with two gRNA-encoding plasmids. **a**) The amplified DNA from cells which were treated with gRNA+100 and gRNA+676 were sequenced using the Venus-Reverse2 primer. The position of targeted deletion is shown with a red triangle. **b**) Alignment of the sequenced DNA with the Venus sequence. Corresponding to the cutting site of gRNA+100 and gRNA+676, the deleted fragment located between nucleotides 95 and 681 of the Venus cDNA.
